# Win for your kin: Neural responses to personal and vicarious rewards when mothers win for their adolescent children

**DOI:** 10.1371/journal.pone.0198663

**Published:** 2018-06-07

**Authors:** Jochem P. Spaans, Sarah M. Burke, Sibel Altikulaç, Barbara R. Braams, Zdeňa A. Op de Macks, Eveline A. Crone

**Affiliations:** 1 Department of Developmental and Educational Psychology, Institute of Psychology, Leiden University, Leiden, the Netherlands; 2 Leiden Institute for Brain and Cognition, Leiden University, Leiden, the Netherlands; 3 Department of Clinical Developmental Psychology, Faculty of Behavioural and Movement Sciences, Institute of Psychology, Vrije Universiteit Amsterdam, Amsterdam, The Netherlands; 4 Department of Psychology and Center for Brain Science, Harvard University, Cambridge, Massachusetts, United States of America; University of Vienna, AUSTRIA

## Abstract

Mother-child relationships change considerably in adolescence, but it is not yet understood how mothers experience vicarious rewards for their adolescent children. In the current study, we investigated neural responses of twenty mothers winning and losing money for their best friend and for their adolescent child in a gambling task. During the task, functional neuroimaging data were acquired. We examined the activation patterns when playing for or winning for self, adolescent children and friends in four a-priori selected ROIs (nucleus accumbens, dorsomedial prefrontal cortex, precuneus and temporo-parietal junction). Behaviorally, mothers indicated that they experienced most enjoyment when they gained money for their children and that their children deserved to win more, relative to friends and self. At the neural level, nucleus accumbens activity was stronger when winning versus losing. This pattern was not only found when playing for self, but also for friends and children, possibly reflecting the rewarding value of vicarious prosocial gains. In addition, dorsomedial prefrontal cortex, precuneus, and temporo-parietal junction were more active when receiving outcomes for children and friends compared to self, possibly reflecting increased recruitment of mentalizing processes. Interestingly, activity in this network was stronger for mothers who indicated that their children and friends deserved to win more. These findings provide initial evidence that vicarious rewards for one’s children are processed similarly as rewards for self, and that activation in social brain regions are related to social closeness.

## 1. Introduction

Mother-child relationships represent a unique social connection which changes during adolescence. While adolescents spend more time with peers and less with their parents [1], they are still dependent on their parents [2,3]. Many studies have shown that developing autonomy from parents while retaining a positive attachment is of key importance for the well-being of adolescents, for instance for fostering the development of peer-relations and the formation of a stable self-image [4–6]. Even though many prior studies have examined the effects of the mother-child relationship on the child [5,7–9], less is known about how mothers themselves experience, and are affected by this relationship once their children reach the age of adolescence. The current study aimed to fill this gap in the literature by investigating neural markers of social connection [10] between mothers and adolescent children, specifically by examining how mothers experience vicarious rewards (rewards received for others) for their children.

Prior studies have found that receiving vicarious rewards for close others, such as friends, is experienced as rewarding, but that personal rewards are experienced as most pleasurable [11]. For personal rewards, it is well known that a broad range of different rewards, such as monetary, social, and food rewards are associated with activation in the ventral striatum [12–14]. Vicarious rewards also result in ventral striatum activity, but only when others are liked or well-known [11,15,16], underlining the important role of relationship closeness in vicarious gaining. Most interestingly, there is evidence that adolescents show highly similar neural activity when gaining vicarious rewards for their mothers [17] and when gaining for themselves [16], in contrast to gaining for someone they dislike [11]. Given the unique nature of the mother-child relationship, an intriguing question is whether this overlap in activation in the ventral striatum during vicarious gaining found in adolescent children is present in mothers as well.

Besides the ventral striatum, brain regions that are associated with mentalizing and perspective taking are also more activated when receiving rewards for others than when receiving rewards for self [16]. In a prior study, Braams et al. [11] demonstrated that the left temporo-parietal junction (TPJ), precuneus, and dorsal medial prefrontal cortex (dmPFC) were active when outcomes were presented for others, relative to self; independent of whether these outcomes were gains or losses. These brain regions are often referred to as the social brain network [18–20], as they are consistently engaged when thinking about the thoughts and actions of other people. Possibly, these brain regions are involved in thinking about others in relation to the self [21,22] or when switching perspectives between self and other [23,24].

Taken together, prior studies showed an important role of the ventral striatum in gaining versus losing for self and others [25], and of the left TPJ, precuneus and dmPFC when thinking about others versus self [16,26], but it is currently not known whether mothers engage these regions when experiencing vicarious rewards for their adolescent children and how activation in these regions is moderated by relationship closeness. Earlier studies have suggested that neural activation in the ventral striatum is dependent on individual differences in relationship closeness [11,15,16,27]. Therefore, it is possible that the relationship between gaining for self and gaining for others is dependent on perceived similarity with the other [28].

In the current study, we explored the neural responses of 20 mothers who gained money for themselves, their best friend, and their adolescent child in a gambling task. Afterwards, we asked them how much their adolescent child and best friend deserved to win and how much they enjoyed winning for their adolescent child and best friend. Participants’ children took part in the same study two years earlier [27]. Based on the results of this prior study, we expected increased neural activity in the ventral striatum, and more specifically in the nucleus accumbens (NAcc) when winning versus losing money for self [25,29]. Therefore, the NAcc was selected as a region of interest in the analyses.

Based on our prior studies in both children and adults, which showed that reward-related brain activation was dependent on social relationship [11], we expected that gaining for friends, and even more so for children, would also result in NAcc activation in mothers [30]. To test for the involvement of social brain regions, we examined activity in the TPJ, precuneus and dmPFC. Since we used the exact same task as in Braams’ design [11], we selected left TPJ, precuneus and dmPFC from this prior study that included young adults as our a priori regions of interest. This allowed us to perform hypothesis-driven analyses with more statistical power than in exploratory whole-brain analyses [31].

Based on studies showing that the strength of a relationship can moderate neural activation during vicarious gaining [11,15,16,27], we also expected to find enhanced activation in left TPJ, precuneus and dmPFC during outcomes for others compared to during outcomes for self, and we hypothesized that this activation would be more pronounced with higher levels of relationships closeness [30,32]. Importantly, given the small sample size of our study (*N* = 20), the results of these tests for individual differences were meant to be exploratory and hypothesis generating, and should be interpreted with this goal in mind.

## 2. Materials and methods

### 2.1. Participants and procedure

Twenty-three female participants between 41 and 55 years of age participated in this study (*M* = 48.17 years, *SD* = 4.33 years). One participant decided to opt out of the fMRI part of the study and was not included in further analyses. After image quality control, two additional participants were excluded from analyses due to significant signal dropout. Thus, the final sample consisted of twenty female participants between 41 and 55 years of age (*M* = 46.80 years, *SD* = 4.14 years). All participants were mothers of children (boys and girls) aged between 13 and 16 years, who were participating in a longitudinal neuroimaging study on adolescent brain development (the Braintime study, see [27]) and in which they performed the same gambling task as reported here. The study and its procedures (protocol NL34234.058.10) were approved by the ethical commission board of the Leiden University Medical Center (local university medical center; reference P10.191/NV/ib). Written informed consent was obtained from all participants on the day of the study. All participants were right-handed and had normal or corrected-to-normal vision (through contact lenses or a set of plastic glasses with adjustable dioptric). Participants were screened for MRI contra-indications and for (history of) neurological and / or psychiatric disorders on three separate occasions (once by phone, once by e-mail and once in person, on the testing-day). All anatomical MRI scans were reviewed by a radiologist. No anomalous findings were reported.

Prior to the testing day, participants filled out online questionnaires from their homes. At the beginning of the FQS questionnaire, participants were asked to nominate their best friend and to fill out the FQS with that friend in mind. The instructions stressed that the nominee had to be a same-sex friend who was not a family member. Nominated friends were not required to attend the scanning session with the participant.

On the testing day, participants were first informed of the study and its procedures, and had the opportunity to (re-)read all detailed study information and to ask questions. Then, after signing the informed consent form, participants shortly practiced the gambling task on a laptop. Next, participants filled out an MRI-checklist to clear them for MRI scanning. Then, we confirmed with the participants on the day of the scanning session that their nominated best friend was a female (same-sex) non family member. In two cases, the participants had no best same-sex friend. One of these two participants nominated her sister and the other her male best friend. Next, the scanning session took place. In addition to the gambling task described in this paper, our scanning protocol consisted of a calibration scan, a resting state scan, a T1 structural scan and diffusion tensor imaging. The entire scanning session lasted approximately 40 minutes. After the scanning session, participants filled out the exit interview questionnaire and the IOS (Inclusion of Other in Self scale). The other questionnaires were filled out through an online survey emailed to the participants a week before the testing day. Finally, participants completed two subscales (similarities and block patterns) of the WAIS-IV (Wechsler Adult Intelligence Scale). All participants received a brain-shaped flash drive, a €30 recompense for participation, and €3 extra won in the gambling task either for themselves, for their child or for their best friend.

### 2.2. Materials

#### 2.2.1. Gambling task

**Fig 1:**
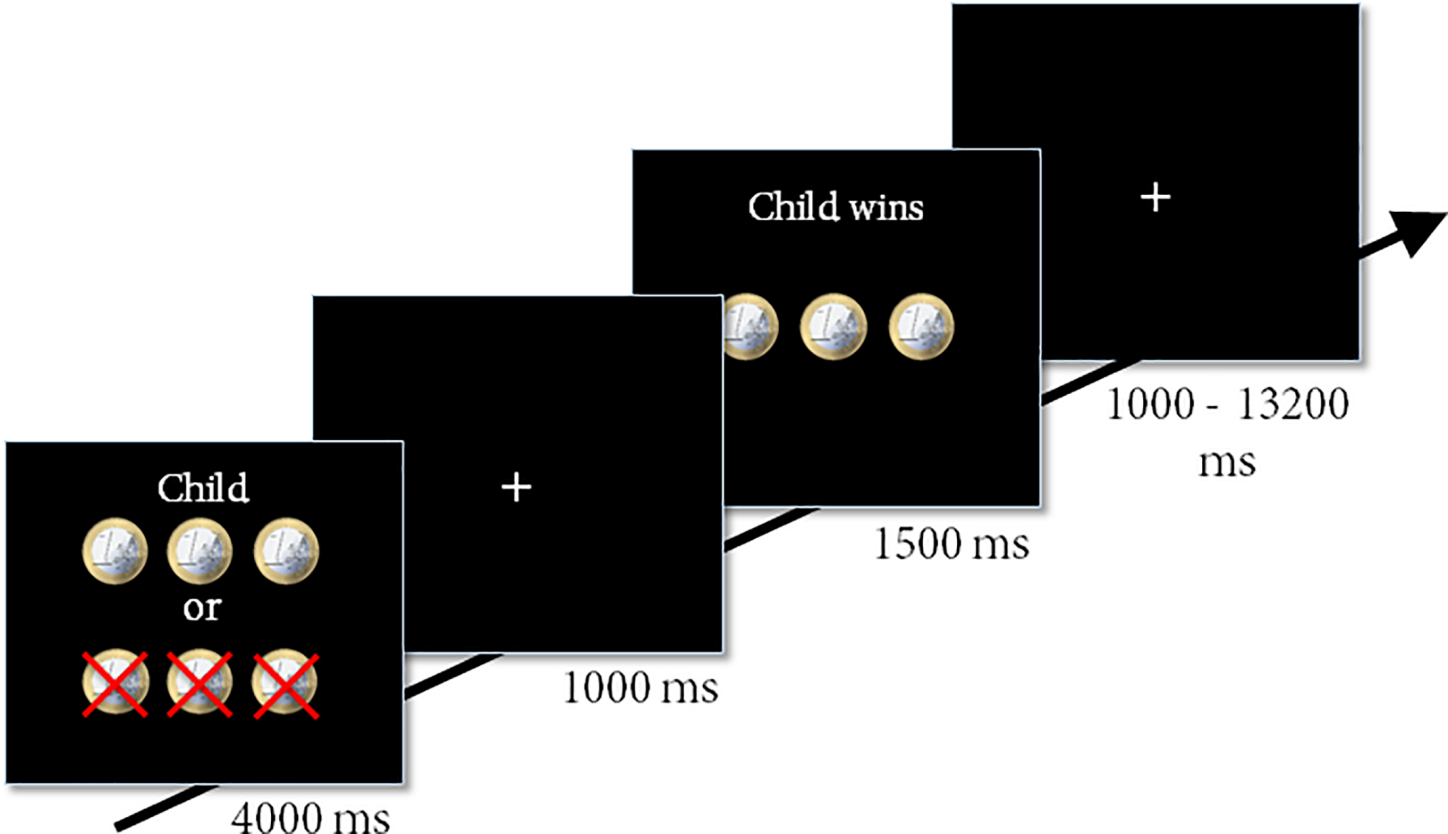
Example trial of the ‘self’ condition. First, participants were informed for whom they would be gambling (self, best friend, or child), and the number of coins that they could win or lose for the respective target. On this screen, participants then chose heads or tails with a left or right button press. After a fixation phase, participants received feedback (gain or loss) about the number of coins that was won or lost for the respective target.

In order to investigate responses to winning and losing for self, best friend and child, participants performed a gambling task (see [11,17,30,33,34]) in which they could repeatedly win or lose money by predicting whether a “coin flip” (with 50/50 odds to win or lose) made by the computer would land heads or tails. If participants correctly predicted the outcome, they won the number of coins that was displayed on the screen for that particular trial. If participants did not correctly predict the outcome, they lost the number of coins that was displayed. There were three possible variations to make the task more engaging: 1) the possibility to win 5 or lose 2 coins; 2) the possibility to win 3 or lose 3 coins and; 3) the possibility to lose 5 or win 2 coins. We collapsed across these trial types in the analyses. Participants played the gambling task for three different targets; for themselves (30 trials), for their best friend (30 trials), and for their child (30 trials), see Fig 1.

Participants completed 90 trials (30 trials for each condition) in two separate fMRI runs, with a short break in between. Each trial lasted 6500 ms with a variable inter-trial interval (jitter) of 1000–13200 ms. The task lasted approximately 13 minutes, excluding the break. We used an event-related design, with jitter durations and trial sequences that were optimized with the program OptSeq2 [35]; see also (http://surfer.nmr.mgh.harvard.edu/optseq/). Heads-choices were indicated by a left button press made with the right index finger; tails-choices were indicated by a right button press made with the right middle finger. Participants were told that at the end of the task one of the trials in the game would be selected at random, and that the target of the selected trial would be paid out the amount of money they won during the game. In actuality, 50% of participants received the pay-out for their best friend and 50% of participants received the pay-out for their child; the money paid was always a fixed amount of € 3.

#### 2.2.2. Gambling task exit interviews

To assess indices of social connection, participants were asked after the scanning session how much they liked winning and losing for themselves, their best friend and their child. In addition, the participants were asked if they believed their best friend and child deserved to win. On all these eight questions, responses were given on a Likert-type scale ranging from 1 ‘not at all’ to 10 ‘very much’.

#### 2.2.3. Interpersonal closeness

As an additional measure of closeness of the relationships between both the participant and her best friend, as well as between the participant and her adolescent child, we used the Inclusion of Other in Self Scale (IOS) [36]. The IOS is a one-item scale on which participants indicate their perceived closeness to another person by selecting one of 7 Venn diagrams with two circles. One of the circles represents the self, and the other represents the other (friend or child). The degree of overlap of the two circles, ranging from 1 (fully separated) to 7 (fully overlapping) provides an index of relationship closeness.

#### 2.2.4. Friendship quality

In order to measure the relationship quality between mother and friend, participants completed the Friendship Quality Scale (FQS) [37] a week before performing the gambling task. The FQS consists of twenty-nine items assessing both positive (eighteen items) and negative (eleven items) friendship quality. An example of a positive quality item is “When I do something well, she is happy for me”; an example of negative quality item is “Sometimes it seems I care more about our friendship than she does”. Participants rated the validity of these statements for their friendship on a five-point Likert? scale ranging from 1 (not true at all) to 5 (very true). The internal consistency of the scale in the current sample (with negative items recoded) was high (Cronbach’s α = .95). For the purposes of this study, we split the Friendship Quality Scale into two variables: one reflecting average positive friendship quality, and one reflecting average negative friendship quality.

#### 2.2.5. Parenting style

We included a measure of parenting style to be able to distinguish between effects of relationship closeness and parenting style. To assess their ratings of their own parenting styles, mothers filled out the parent-report version of the EMBU (Egna Minnen av Barndoms Uppfostran–Own memories of child’s upbringing); [38] specifically a Dutch translation of the 24-item version of the EMBU-P that has been shown to have excellent validity in an adolescent sample [39]. This version of the EMBU-P consists of three subscales; emotional warmth, rejection, and overprotection. An example item of the emotional warmth subscale is “You respect your child’s opinions”. An example item of the rejection subscale is “You punish your child even for small offences”. An example item of the overprotection subscale is “When your child comes back home, he/she always has to account for what he/she has been doing”. Responses were given on a Likert-type scale ranging from 1 to 4 (1 = never, 2 = sometimes, 3 = often, 4 = almost always).

### 2.3. MRI data acquisition

MRI data were collected using a Philips 3.0 Tesla scanner with a standard eight channel whole-head coil using non-parallel image techniques. For functional MRI scans, we used T2*- weighted Echo-Planar Imaging (TR = 2.2s, TE = 30 ms, FOV: 220 mm, 80 x 80 matrix, 2.75 mm in-plane resolution). Functional scans consisted of 2 runs with 175 and 169 volumes respectively. Participants were able to see a screen on which the task was projected through a mirror attached to the coil. In addition to fMRI sequences, we collected structural images for anatomical reference (high resolution 3D T1-weigthed), TR = 9.751 ms, TE = 4.59 ms, FOV = 224 x 168 x 177 mm. Participants’ head movements were restricted by using foam cushions inserts.

### 2.4. MRI data analysis

#### 2.4.1. Preprocessing

We used SPM8 (Statistical Parametric Mapping; Wellcome Trust Centre for Neuroimaging, London, UK) to preprocess and analyze MRI data. During preprocessing, we first corrected all images for motion and slice timing acquisition, followed by registering the functional images to the individual anatomical image, after which we spatially normalized the functional scans to T1 templates which were based on the MNI305 stereotaxic space [40]. Images were not segmented into grey matter, white matter and cerebrospinal fluid (csf). All volumes were resampled to voxels of 3x3x3 mm. Finally, we used a 6 mm full width half maximum isotropic Gaussian kernel to spatially smooth the data. Non-brain tissue was masked out for functional analyses.

#### 2.4.2. fMRI-analysis

To calculate the relevant contrasts, we modeled the fMRI time series convolved with the hemodynamic response function (HRF) with events that corresponded to the stimulus (i.e. when participants were shown the target and the number of coins on stake for the current trial) and outcome phases of a trial (i.e. when feedback about the amount won or lost was given). The time series were modeled as a zero-duration function at stimulus onset, and for the full duration of the outcome (1500 ms). Trials with no response from the participants were modeled separately as invalid trials, and were not included in further contrasts. This occurred in 0.3% of the trials.

The modeled events were added as regressors in a general linear model, along with a basic set of cosine functions that high-pass filtered the data (with a high pass-cutoff of 120 seconds) and a covariate for run effects. Based on the image realignment process, individual head jerks (> 1 mm displacement) were identified and—together with the six motion parameters—included as nuisance variables in every first-level design matrix [41] to account for the effects of excessive head motion. The least squares parameter estimates of height of the best-fitting canonical HRF for each condition were used in pairwise contrasts.

#### 2.4.3. fMRI region-of-interest analysis

We focused our analyses on four pre-defined ROIs (NAcc, left TPJ, precuneus, and dmPFC) that were shown to be involved in reward processing of personal and vicarious rewards in the same gambling task in previous research [11]. We used the Marsbar toolbox in SPM for extracting activation parameters from the regions of interest [42].

We used a bilateral predefined anatomical NAcc mask (total volume 1408 mm^3^) that was extracted from the Harvard-Oxford subcortical atlas and thresholded at 40%. The mask consisted of 28 voxels for the left NAcc (coordinates left: x = -9.57, y = 11.70, z = -7.10) and of 26 voxels for the right NAcc (coordinates right: x = 9.45, y = 12.60, z = -6.69). The final analyses were performed collapsed across left and right NAcc. The masks for the left TPJ, precuneus, and dmPFC were based on the regions found to be selectively involved when playing for others rather than for self [11]. Therefore, spheres with an 8 mm radius around the peak level of activation as reported in Braams et al. [30] were defined as regions of interest in the current study (TPJ: x = -48, y = -63, z = 39; precuneus: x = -3, y = -60, z = 33; dmPFC: x = -9, y = 51, z = 36).

#### 2.4.4. Brain behavior correlations

To assess whether the brain activity patterns were related to the behavioral measures, we related activation in our predefined ROIs to 1) the extent to which mothers indicated to like winning for self, friend and child, and 2) the extent to which mothers believed their friends or children deserved to win, 3) relationship closeness, as indicated on the IOS scales, and 4) parenting style as measured by the EMBU-P. For the NAcc ROI we computed a win-lose difference score of activation levels for each target, since we did not expect differences between activation for wins and losses for the precuneus, dmPFC and left TPJ ROIs. Therefore, we computed difference scores for outcomes for friend versus outcomes for self (friend–self), and outcomes for child versus outcomes for self (child–self) that were collapsed across wins and losses. Thus, the first set of difference scores (for the NAcc) reflected the relative value of outcomes for each target, and the second set of difference scores (for the precuneus, dmPFC and left TPJ) reflected activation that was stronger in thinking about outcomes for friend or child than when thinking about own outcomes, regardless of outcome-valence.

Before we computed correlations between our ROI data and behavioral measures, we first assessed outliers in the behavioural variables. To do this, we standardized our data and looked for cases with z-scores lower than -3.2 and / or higher than 3.2. Although there were no cases meeting these criteria, there were 4 cases with z-scores higher than 2 or lower than– 2. For these 4 cases, we inspected both Cook’s distances and leverage values. None of Cook’s distances were larger than 1, and all leverage values were .05. Based on these results, we chose not to exclude any additional cases (in addition to the cases that were removed due to signal-dropout that were mentioned earlier).

## 3. Results and discussion

### 3.1. Self-report data

#### 3.1.1. Descriptive statistics

Descriptive statistics for each of the subscales of the parenting scale (EMBU; possible values 1–4) and the FQS (possible values 1–5) are presented in Table 1.

**Table 1:** EMBU-P and FQS.

	Min.	Max.	*M*	*SE*	*SD*
**EMBU Emotional Warmth**	2.63	4.00	3.58	.08	.34
**EMBU Overprotection**	1.25	2.63	1.87	.08	.34
**EMBU Rejection**	1.00	1.88	1.29	.06	.25
**Negative Friendship Quality**	1.00	2.36	1.30	.08	.34
**Positive Friendship Quality**	1.00	4.89	3.95	.25	1.13

Descriptive statistics of the EMBU and FQS questionnaire subscales. Minimum and maximum values, means (*M*), standard errors (*SE*) and standard deviations (*SD*) are displayed.

#### 3.1.2. Inclusion of other in self-scale & exit interview variables

On the IOS (scale 1–7, with 7 referring to closest connection), a Wilcoxon Signed-Ranks Test showed that the median ranks for mothers’ relationship with child (*Mdn* = 5.5) were significantly higher than the median ranks for relationship with their best friend (*Mdn* = 4), *Z* = 3.35, *p* = .001, *r* = .22.

Next, we performed a non-parametric Friedman test of differences among repeated measures to investigate differences between winning enjoyment for different targets (“Self-Win enjoyment”, “Friend-Win enjoyment”, and “Child-Win enjoyment”, answers ranged between 1–10). Results showed that there was a significant difference between targets, Χ^2^ (2, 20) = 27.72, *p* < .001. Subsequent pairwise comparisons for enjoyment ratings using Wilcoxon Signed-ranks Tests resulted in significantly higher median ranks for Child (*Mdn* = 9.0) compared to Self (*Mdn* = 6.5), *Z* = 3.56, *p* < .001, *r* = .79, and higher median ranks for Friend (*Mdn* = 7.0) compared to Self, *Z* = 2.24, *p* = .025, *r* = .50. In addition, the median ranks of enjoyment ratings were significantly higher for Child than for Friend, *Z* = 3.59, *p <* .001, *r* = .80. Finally, a Wilcoxon Signed-ranks Test for deserving to win scores (“Friend deserved win” and “Child deserved win”) revealed that mothers indicated that their children (*Mdn* = 9.0) deserved to win more than their best friends (*Mdn* = 8.35), *Z* = 2.04, *p* = .041, *r* = .46.

Table 2 shows Spearman correlations between the exit interview self-report variables. There were significant correlations between “Friend-win enjoyment” and “Child-win enjoyment”, and between “Friend deserved win” and “Child deserved win”. In addition, there was a significant correlation between “Inclusion of friend in self” and “Inclusion of child in self”, as measured with the IOS.

**Table 2:** Correlations between exit interview variables and inclusion of other in self.

Variable	1.	2.	3.	4.	5.	6.
**1. Self-win enjoyment**	_					
**2. Friend-win enjoyment**	.34	_				
**3. Friend deserved win**	-.39	.36	_			
**4. Inclusion of friend in self**	.19	.39	-.02	_		
**5. Child-win enjoyment**	.31	.86***	.34	.28	_	
**6. Child deserved win**	-.17	.37	.70***	-.15	.44	_
**7. Inclusion of child in self**	.38	.41	-.15	.78***	.33	-.9

*** Correlation is significant at the .001 level (two-tailed)

### 3.2. Regions of interest activation during gambling task

To test the roles of activation in our predefined regions of interest for winning and losing for different targets and outcomes, we performed 3 (Target: Self, Friend, Child) x 2 (Outcome: Win, Loss) repeated-measures ANOVAs for each of the 4 ROIs.

#### 3.2.1. NAcc

**Fig 2:**
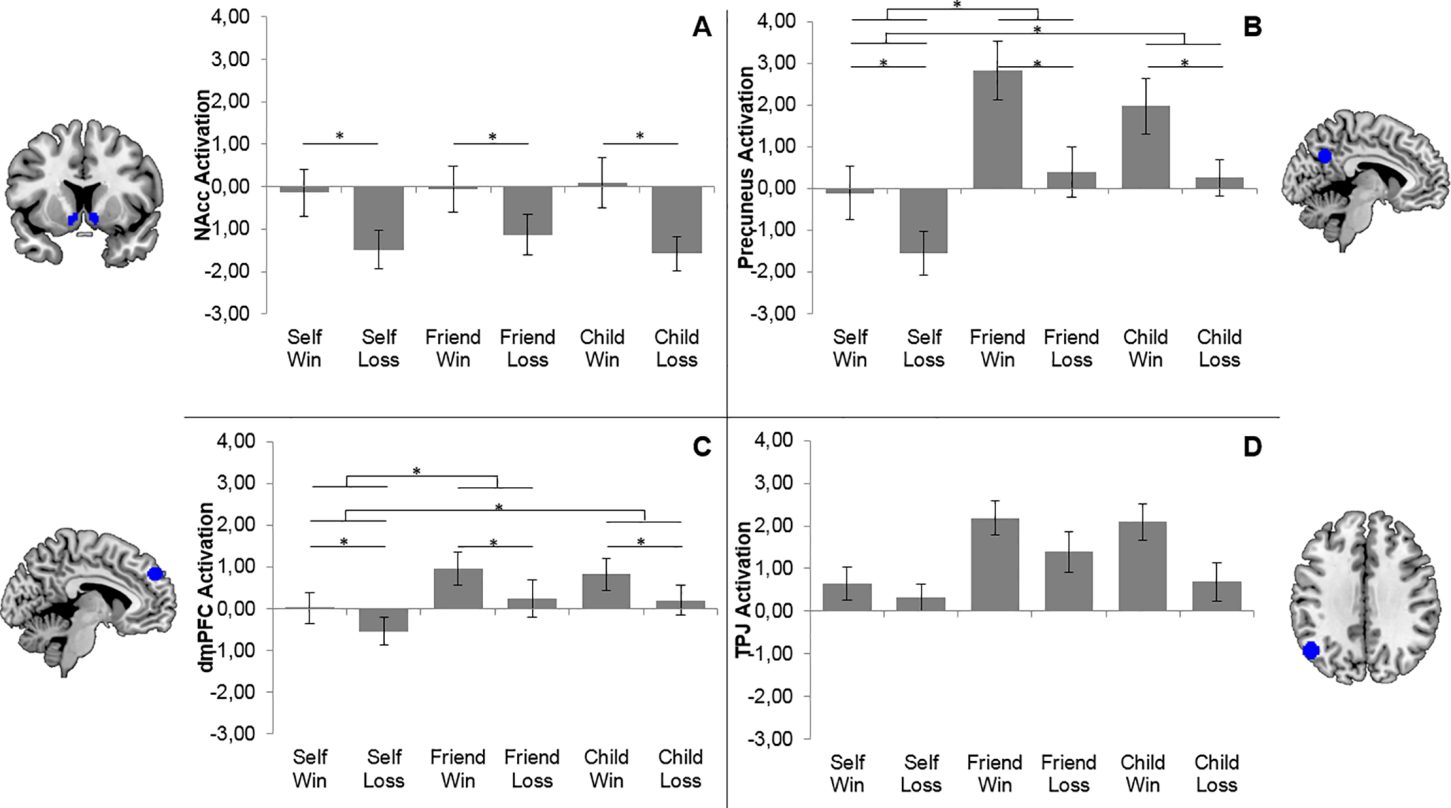
Activation in all contrasts versus fixation for ROIs in the NAcc (A), Precuneus (B), dmPFC (C) and left TPJ (D). Differences flagged with * and ** are significant with respective alphas .05 and .01. All activations are presented relative to the jittered inter-trial fixation baseline.

The ANOVA for NAcc resulted in a main effect of Outcome (*F*(1,19) = 39.40, *p* < .001), but no main effect of Target or interaction effect with Target. As can be seen in Fig 2A, NAcc activation was higher for Wins than for Losses for all targets (Self, Friend and Child).

#### 3.2.2. Precuneus

The ANOVA for the precuneus resulted in a main effect of Outcome (*F*(1,19) = 44.83, *p* < .001), and a main effect of Target (*F*(2,38) = 66.55, *p* < .001), but no Target x Outcome interaction. As can be seen in Fig 2B, Wins resulted in more activity than Losses, and post hoc paired comparisons revealed that outcomes for Friend and Child resulted in more activity in the precuneus than outcomes for Self (*t*(19) = 4.30, *p* < .001 and *t*(19) = 5.92, *p* < .001). Friend and Child did not differ from each other (*t*(19) = 1.26, *p* = .22).

#### 3.2.3. dmPFC

The ANOVA for the dmPFC resulted in a main effect of Outcome (*F*(1,19) = 12.07, *p* < .05) and a main effect of Target (*F*(2,38) = 3.91, *p* < .05). There was no significant interaction between Outcome and Target. As can be seen in Fig 2C, Wins resulted in more activity than Losses. Pairwise comparisons showed that activations related to both Friend and Child were stronger than activations related to Self (respectively *t*(19) = 4.25, *p* < .001 and *t*(19) = 3.51, *p* < .01), but Friend and Child did not differ from each other (*t*(19) = 1.74, *p* = .10).

#### 3.2.4. TPJ

The ANOVA for left TPJ resulted in non-significant main effects of Outcome (*F*(1,19) = 9.92, *p* = .05) and Target (*F*(2,38) = 12.58, *p* = .06) (see Fig 2D). Although these effects were not statistically significant, both effects were very close to significance and were found in the expected direction. There was no significant interaction effect (*p* = .35).

### 3.3. Brain-behavior correlations

#### 3.3.1. Correlations with enjoyment and deserving ratings

First, we investigated Spearman correlations between the Win-Lose contrast for NAcc for each target, and a difference score of the win-lose enjoyment exit questions for each target. Results showed that there was a significant correlation between the activation in the Win-Lose contrast for Self and the difference score for win-lose enjoyment exit question for Self (*ρ*(20) = .51), *p =* .023). Neither of the correlations between activation in this contrast and the win-lose enjoyment exit questionnaires for Child and Friend were significant (*p* = .757 and *p* = .421, respectively).

Next, we investigated Spearman correlations between activation in Friend-Self and Child-Self contrasts in TPJ, dmPFC and precuneus, and winning enjoyment. These analyses were performed based on general target activation, collapsed across Win and Loss outcomes. For this purpose, we computed the difference scores Friend-Self and Child-Self for neural activity, and the difference scores Friend-Self winning enjoyment and Child-Self winning enjoyment for the exit questionnaire items. Results showed no significant correlations between activation in any of the Friend-Self or Child-Self contrasts (TPJ, precuneus, and dmPFC) and the respective winning-enjoyment variables (*p*-values ranged between .064 and .676, two-tailed).

**Fig 3:**
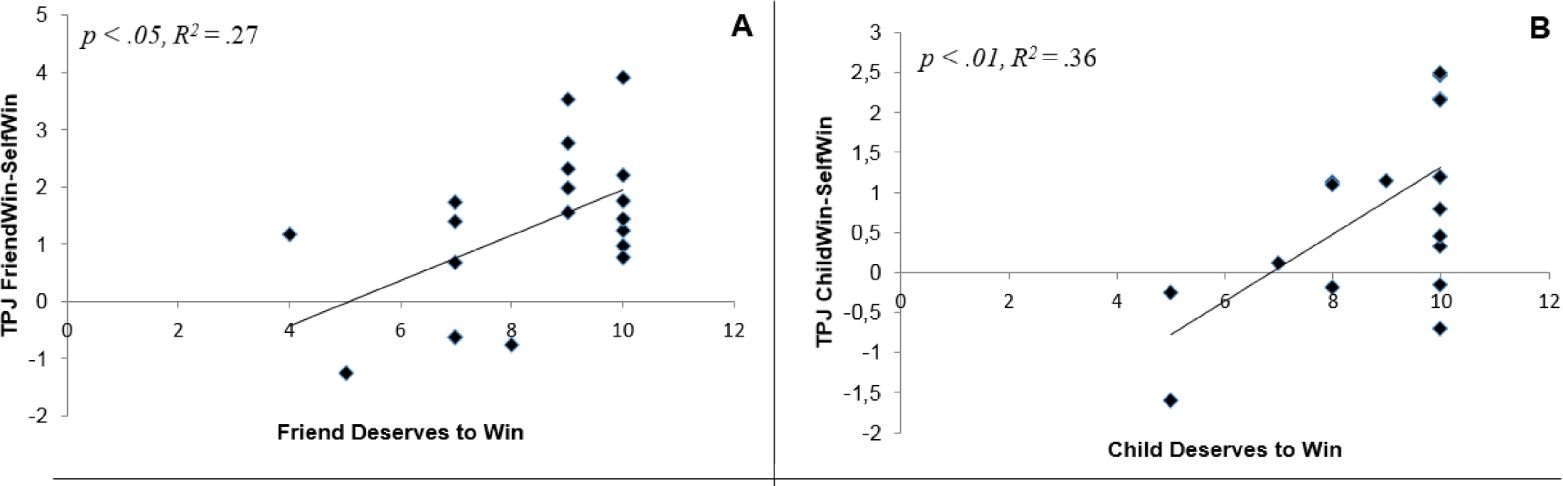
A) A difference score of activation in TPJ in the FriendWin–SelfWin contrast is plotted on the y-axis. The degree to which participants indicated that their friend deserved to win is plotted on the x-axis. B) Difference scores of activation in TPJ in the ChildWin–SelfWin contrasts is plotted on the y-axis. The degree to which a participant indicated that their child deserved to win is plotted on the x-axis.

The same analyses were performed for the exit question “deserves to win” for Friend and Child respectively. Results showed that activation in TPJ for the Friend-Self contrast was positively correlated with the “Friend deserves to win” variable, *ρ*(20) = .46, *p =* .039 (two-tailed) (see Fig 3A). These findings indicate that the more participants reported that they thought their friend deserved to win, the more TPJ activation was found in friend-trials compared to self-trials (irrespective of outcome). Similar to the friend condition, we found significant positive correlations between activation in TPJ (Fig 3B), in the contrast Child-Self and the “Child deserved to win” variable, *ρ*(20) = .55, *p =* .012 (two-tailed). That is, the more participants indicated that their child deserved to win, the more activation was found in the TPJ during child-trials compared to during self-trials. We found no significant correlations between the contrasts for the dmPFC and precuneus and deserving to win (*p*-values ranged between .093 and .637, two-tailed).

#### 3.3.2. Correlations with inclusion of other in self and parenting style

Another set of Spearman correlations was computed between the ROI contrasts for Self-Friend and Self-Child, and Inclusion of friend and child in self. There were no significant correlations (*p*-values ranged between .278 and .933).

#### 3.3.3. Correlations with parenting style

Finally, we computed Spearman correlations between the ROI contrasts for Self-Friend and EMBU-P subscales. There were no significant correlations (*p*-values ranged between .081 and .995).

### 3.4. Discussion

The goal of this study was to examine how mothers process vicarious winning for their adolescent children in terms of behavioral ratings and neural measures. For this purpose, we compared responses to gaining monetary rewards for self, and vicarious gaining of monetary rewards for adolescent children using a previously validated vicarious gambling task [30]. To examine responses for other, egalitarian close relations, we also included a best friend condition [27]. The study resulted in several important findings. First, mothers felt closer to their adolescent children than to their best friends, and felt that their children deserved to win more than their friends. Furthermore, they experienced winning for their children as more pleasurable than winning for their friend, and winning for friends was experienced as more pleasurable than winning for self, possibly reflecting general prosocial tendencies. Interestingly, participants showed similar NAcc responses when winning for themselves, their friends and their adolescent children. The effects of individual differences between mothers on neural activation during vicarious gaining were most apparent in social brain regions. That is, we found that neural activation in the TPJ when mothers were playing for their child and friend, was stronger when mothers indicated that they thought their friend or adolescent child deserved to win more. The discussion is organized according to the neuroimaging findings.

#### 3.4.1. Vicarious rewards in NAcc

Prior studies have demonstrated the importance of the ventral striatum when gaining for self and others [13,25]. A meta-analysis revealed that the striatum is generally more active when we gain for ourselves relative to others, suggesting that the ventral striatum represents a basic self-relevant reward value [13,29]. Vicarious reward responses in the ventral striatum, however, are dependent on the beneficiary, and have only been observed in vicarious gaining for close relations [15,16]. Mothers in the current study indicated by self-report that they enjoyed winning for friends and their adolescent children more than for themselves. However, winning vicarious rewards for close others (friends and their adolescent children) resulted in similar NAcc activation as personal rewards. These findings highlight the importance of closeness in experiencing vicarious rewards, given that prior studies suggested that NAcc only responds to personal rewards for close others and not for distant others [11,16]. There was no evidence for stronger activity for vicarious rewards for children than for self (as was suggested by the self-report outcomes). Thus, even though the self-reports showed that winning enjoyment was higher for children than for friends, this was not observed in neural activation, which was similar when gaining for friends and children. It is currently unclear what causes the differences between self-report findings and neural activation. Possibly, NAcc represents more basic reward experiences, or possibly self-report measures used to gauge winning enjoyment are biased by social norms. Future research using larger samples is necessary to investigate this finding more thoroughly.

#### 3.4.2. Social brain activity

An additional question that was addressed was the role of social brain regions, specifically dmPFC, left TPJ and precuneus, when receiving outcomes for self and others. The current findings are consistent with prior research showing that regions within the social brain network are more active when receiving outcomes for others (friends and children) compared to receiving outcomes for themselves [16,30]. Even though in general these regions were more engaged for winning than losing, there was no interaction with target, suggesting that these regions generally process outcomes for close others. Most mothers reported that they enjoyed winning for their friends and children and that they believed their friends and children deserved to win (here referred to as social closeness), but there were also individual differences in the extent of social closeness. Interestingly, TPJ activation levels, when receiving outcomes for children and friends, were consistently related to individual differences in whether mothers thought that their adolescent child or their friend deserved to win. That is, mothers who indicated that they thought their child or friend deserved to win more showed stronger activity in these three regions when playing for their child or friend relative to playing for themselves. Since previous studies have underlined the importance of the TPJ in mentalizing processes [18,43], these patterns of activation in the current study could reflect heightened engagement in mentalizing as a function of social connection to the target for whom they were playing.

Previous studies have suggested that socially warm exchanges may be associated with neural responses when gaining for others [44]. Moreover, activity in social brain regions is associated with social bonds with friends in adolescence, especially with whether the adolescent thinks the friend deserves to win [17]. The current findings confirm that similar relations are observed for mothers, both when they win for their child and when they win for their best friend. Future research should test this question in more detail, but possibly the question whether the child or friend deserves to win reflects a process that is engaging the social brain network more generally [26,45].

#### 3.4.3. Limitations

This study has several limitations. First, the sample size of this study is small (*N* = 20). For this reason, we tested specific hypotheses in a priori defined regions of interest that were based on a separate study that used a similar paradigm and did not rely on exploratory whole brain analyses. As a consequence of the small sample size, the tests for individual differences should be interpreted with caution and with the goal to be exploratory and hypothesis-generating. Future studies are needed to confirm these findings in larger sample sizes. Second, the inclusion of mothers occurred two years after the inclusion of the children, which limits the possibility to test for direct mother-child neural synchrony. Third, the current paradigm included only win and lose trials, without a no-win baseline. Future studies should include this baseline to test for relations with ventral striatum activity in more detail. Finally, in the current design, it is not possible in the IOS (inclusion of other in self) scale to distinguish whether stronger relations with children or best friends found between individuals are the result of individual differences in a general tendency to feel closer to others or due to individual differences in relationship closeness to child and to best friend. Because the IOS lacks a baseline, we cannot distinguish between participants who generally feel closer or more distant to others. Future designs using the IOS benefit from including a ‘stranger’ item, to obtain a reference point of how close participants feel to others in general.

## 4.1 Conclusions

Taken together, this is a first study to examine vicarious-reward-processing in mothers gaining rewards for their adolescent child and a close other with whom they have an egalitarian relationship (i.e., their best friend). The study confirmed that the ventral striatum is involved in gaining for self as well as for close others [16]. Furthermore, the social closeness to the child, here measured by winning enjoyment and by whether the child deserves to win, was partly related to activity in the TPJ, a part of the social brain network [18]. This study opens several new avenues for future research, such as examining the extent to which adolescents and parents show similar neural activity when gaining for each other, which may be a reflection of relationship strength [28]. The individual difference measures should be interpreted as exploratory, but they provide several intriguing hypotheses about how neuroimaging methods can inform us on mother-child relationships.

## Supporting information

S1 File Dataset. Anonymized dataset that was used for the current study.
(XLSX)

S2 File Parametric analyses. Originally, we conducted parametric analyses to investigate brain behavior relations, and differences between targets on self-reported winning enjoyment and IOS (inclusion of other in self). The results of these analyses can be found in this supporting file.
(DOCX)
