# Supplementary material for: Win for your kin: Neural responses to personal and vicarious rewards when mothers win for their adolescent children
Source: PLoS One. 2018 Jun 7;13(6):e0198663. doi: 10.1371/journal.pone.0198663 (PMC5991740; doi:10.1371/journal.pone.0198663)
Supplement: S2 File — Originally, we conducted parametric analyses to investigate brain behavior relations, and differences between targets on self-reported winning enjoyment and IOS (inclusion of other in self). The results of these analyses can be found in this supporting file. (DOCX) [file pone.0198663.s002.docx]

**S1 File. Supplementary material: parametric tests**

**3.1.2. Inclusion of other in self-scale & exit interview variables**.

On the IOS, mothers indicated that the relationship with their child (*M* = 5.15, *SD* = .38) was significantly closer than with their best friend (*M* = 3.85, *SD* = .38) (scale 1-7, with 7 referring to closest connection), as revealed by a two-tailed independent samples t-test (*t*(19) = 4.95, *p* < .001).

Next, we performed one-way repeated-measures ANOVAs to compare the effect of Target on winning enjoyment (3 levels: “Self-Win enjoyment”, “Friend-Win enjoyment”, and “Child-Win enjoyment”). There was a significant main effect of Target (*F*(1.41, 26.83) = 25.35, *p* < 0.001). Subsequent pairwise comparisons for enjoyment ratings resulted in significantly higher ratings for Child (*M* = 8.65, *SD* = 1.66) compared to Self (*M* = 6.5, *SD* = 1.47) (*t*(19) = 5.57, *p* < .001), and higher ratings for Friend (*M* = 7.25, *SD* = .29) compared to Self (*t*(19) = 2.52, , *p* < .001). In addition, ratings were significantly higher for Child than for Friend (*t*(19) = 6.67, *p* <.001). Finally, a paired sample t-test for deserving to win scores (“Friend deserved win” and “Child deserved win”) revealed that mothers indicated that their children deserved to win more than their best friends (paired t-test, one-tailed, *t*(19) = 1.99, *p* = .03).

Table 2 shows the correlations between the exit interview self-report variables. There were significant correlations between “Self-win enjoyment” and “Friend-win enjoyment”, between “Friend-win enjoyment” and “Child-win enjoyment”, and between “Friend deserved win” and “Child deserved win”. In addition, there was a significant correlation between “Inclusion of friend in self” and “Inclusion of child in self”, as measured with the IOS.

*Table 2.* Correlations between exit interview variables and Inclusion of Other in Self

| **Variable** | **1.** | **2.** | **3.** | **4.** | **5.** | **6.** |
| --- | --- | --- | --- | --- | --- | --- |
| 1. Self-win enjoyment | _ |  |  |  |  |  |
| 2. Friend-win enjoyment | .54^*^ | _ |  |  |  |  |
| 3. Friend deserved win | -.27 | .28 | _ |  |  |  |
| 4. Inclusion of friend in self | .18 | .35 | -.02 | _ |  |  |
| 5. Child-win enjoyment | .40 | .83^*^ | .29 | .28 | _ |  |
| 6. Child deserved win | -.13 | .27 | .64^*^ | -.08 | .36 | _ |
| 7. Inclusion of child in self | .43 | .38 | -.10 | .76^*^ | .39 | .13 |

* Correlation is significant at the 0.01 level (two-tailed)

**3.3. Brain-behavior correlations**

**3.3.1. Correlations with enjoyment and deserving ratings**

First, we investigated Spearman correlations between the Win-Lose contrast for NAcc for each target, and a difference score of the win-lose enjoyment exit questions for each target. There was a significant correlation between the activation in the Win-Lose contrast for Self and the difference score for win-lose enjoyment exit question for Self (*r* = .516, *p <* 0.05). Neither of the correlations between activation in this contrast and the win-lose enjoyment exit questionnaires for Child and Friend were significant (*p* = .178 and *p* = .135, respectively).

Next, we investigated correlations between activation in Friend-Self and Child-Self contrasts in TPJ, dmPFC and precuneus, and winning enjoyment. These analyses were performed based on general target activation, collapsed across Win and Loss outcomes. For this purpose, we computed the difference scores Friend-Self and Child-Self for neural activity, and the difference scores Friend-Self winning enjoyment and Child-Self winning enjoyment for the exit questionnaire items.

Activation in the TPJ for the Friend-Self contrast was significantly positively correlated with the difference score Friend-Self winning enjoyment, *r* = .443 *p* < .05 (one-tailed). A similar pattern was found for the Child-Self contrast. That is, activation in the TPJ for the Child-Self contrast was significantly positively correlated with the difference scores between Child-Self winning enjoyment, *r* = .396 *p* < .05 (one-tailed). There were no other significant correlations between the win-lose difference scores and activation in the Friend-Self or Child-self contrasts (*p*-values ranged between .055 and .950).

The same analyses were performed for the exit question “deserves to win” for Friend and Child respectively. Results showed that activation in TPJ for the FriendWin-SelfWin contrast was positively correlated with the “Friend deserves to win” variable, *r* = .522 *p* < .01 (one-tailed) (see Fig 3A). These findings indicate that the more participants reported that they thought their friend deserved to win, the more TPJ activation was found in friend-trials compared to self-trials (irrespective of outcome). Similar to the friend condition, we found significant positive correlations between activation in precuneus (Fig 3B), dmPFC (Fig 3C), and TPJ (Fig 3D), in the contrast Child-Self and the “Child deserved to win” variable (precuneus: *r* = .405 *p* < .05; dmPFC: *r* = .466, *p* < .05; TPJ: *r* = .602, *p* < .01). That is, the more participants indicated that their child deserved to win, the more activation was found in the TPJ, the precuneus, and in the dmPFC during child-trials compared to during self-trials.

**3.3.2. Correlations with Inclusion of Other in Self and Parenting Style**

Another set of correlations was computed between the ROI contrasts for Self-Friend and Self-Child, and Inclusion of friend and child in self. There were no significant correlations (*p*-values ranged between .160 and .854).

**3.3.3. Correlations with Parenting Style**

Finally, we computed correlations between the ROI contrasts for Self-Friend and EMBU-P subscales. There were no significant correlations (*p*-values ranged between .056 and .459).
